# Dataset of Fourier transform infrared (FTIR) spectra of a primary amine, selected aldehydes, and their Schiff bases acquired using the Invenio-R spectrometer

**DOI:** 10.1016/j.dib.2025.111741

**Published:** 2025-05-31

**Authors:** Mohd Rashidi Abdull Manap, Umar Sani, Musbahu Yahaya, Ahmad Zaidi Ismail, Qhurratul Aina Kholili, Fatin Abu Hasan, Siti Nurul Ain Md Jamil, Reda Dzingelevičienė, Saiful Saiful, Giorgio S. Senesi, Herculano Martinho

**Affiliations:** a Department of Chemistry, Faculty of Science, Universiti Putra Malaysia, 43400 Serdang, Selangor, Malaysia; b Medical Microspectroscopy Research Group, Department of Experimental Medical Science. Lund University, 22180 Lund, Sweden; c Department Pure and Industrial Chemistry, Faculty of Physical Science, Beyero University Kano, Nigeria; d Department of Chemistry, Faculty of Natural and Applied Sciences, Sule Lamido University Kafin Hausa, Jigawa, Nigeria; e Marine Research Institute, Faculty of Health Sciences, Klaipeda University, 92294 Klaipeda, Lithuania; f Department of Chemistry, Faculty of Math and Natural Sciences, Universitas Syiah Kuala, Darussalam-Banda Aceh 23111, Indonesia; g CNR - Institute for Plasma Science and Technology (ISTP) – Bari seat, 70126 Bari, Italia; h Federal University of ABC (UFABC), Av. dos Estados, 5001, 09210-580 Santo Andre-SP, Brazil

**Keywords:** Aldehydes, ATR-FTIR, Imines, Primary amine, Schiff base

## Abstract

This dataset reports on the Fourier-transform infrared (FTIR) spectroscopic analysis of a primary amine and some aldehydes along their corresponding Schiff bases. The dataset presented in this work consists of spectral FTIR data acquired in the 80–6000 cm⁻¹ range, along with the corresponding relative IR intensities of the samples. The primary amine and aldehydes were of high purity and were used to synthesise the corresponding Schiff bases. The FTIR spectra were acquired for the vibrational assignment associated with the samples. The IR spectra were acquired using a Bruker Invenio-R (Universiti Putra Malaysia, Malaysia) spectrometer equipped with attenuated total reflection (ATR) diamond crystal with an accumulation of 64 scans at a spectral resolution of 4 cm⁻¹ and processed using OPUS 8.7.41 software. The data indicated that FTIR spectroscopy can be a powerful tool for analysing Schiff bases.

Specifications Table

**Table:** 

Subject	Spectroscopy
Specific subject area	Fourier Transform Infrared (FTIR)
Type of data	Figures with peak labelling. Raw data in .0 format Experimental file in .xpm format
Data collection	FTIR spectra were acquired employing a Bruker Invenio-R (Universiti Putra Malaysia) spectrometer equipped with attenuated total reflection (ATR) diamond with an accumulation of 64 scans at a spectral resolution of 4 cm⁻¹, and data processing was performed using OPUS 8.7.41 software.
Data source location	Institution: Department of Chemistry, Faculty of Science, Universiti Putra Malaysia Country: Malaysia. Latitude and longitude and GPS coordinates, for collected samples/data: 2°59′28.7"N 101°42′29.5"E
Data accessibility	Repository name: Mendeley data Direct URL to data: Manap, Mohd Rashidi Abdull; Musbahu Yahaya (2024), “Infrared spectra of primary amine and some aldehydes with their corresponding Schiff base obtained using Invenio-R spectrometer”, Mendeley Data, V1 Data identification number: 10.17632/6bfs55tvdh.1 Direct URL to data: https://data.mendeley.com/datasets/6bfs55tvdh/1 Instructions for accessing these data: The spectra are provided in the Opus format. Click on the appropriate URL above to download the .0 file.

## 1 Value of the Data

• The data may be used for the characterization of aldehydes featuring wider IR spectral regions.
• Experimental data are useful for the validation of vibrational spectra with DFT calculations in solid and gas phases.
• This data may serve as a valuable reference for researchers engaged in the synthesis of Schiff bases and their metal complexes, particularly for comparative analysis and characterization studies.

## 2 Background

In Schiff base synthesis, the selection of starting compounds significantly influences the efficiency, biological activity, and structural diversity of the resulting products. Among these, 2-aminothiophenol is widely used due to its ability to impart antimicrobial and antioxidant properties to Schiff bases, with an efficient and straightforward synthesis route [1]. Salicylaldehyde is another versatile precursor that allows for the synthesis of Schiff bases with diverse biological activities, benefits from environmentally friendly synthesis conditions, and enables the study of tautomeric equilibria, making it valuable in both fundamental and applied research [[2], [3], [4], [5]]. Similarly, 2-methoxybenzaldehyde enhances the biological potential of Schiff bases, particularly for antibacterial applications, while offering efficient synthesis with high yields and simple purification processes [2,6]. 2-hydroxy-1-naphthaldehyde is notable for its large conjugated system, which provides Schiff bases with distinct UV/Vis spectral features useful for electronic and structural studies, alongside supporting investigations on tautomerism and solvent interactions [5,7,8]. The incorporation of 2-pyridinecarboxaldehyde introduces an additional coordination site through the pyridine nitrogen, making it highly relevant in pharmaceutical research and metal complexation, with synthesis methods that are both efficient and eco-friendly [4]. Lastly, 2-thiophenecarboxaldehyde, with its sulfur-containing thiophene ring, enhances the electronic properties of Schiff bases and has been shown to yield products with significant antimicrobial activity through practical and cost-effective synthesis approaches [1]. Collectively, these starting materials are strategically chosen for their ability to contribute specific functional properties, making them indispensable in the design of Schiff bases for applications in medicinal chemistry, coordination chemistry, and materials science (Fig. 4).

This dataset [9] was obtained from both a primary amine and a variety of aldehydes, and their corresponding Schiff bases. Samples of primary amine and aldehydes were obtained directly from Sigma Aldrich, while the other Schiff bases were synthesized through a condensation reaction of the amine with the aldehydes. The authors will utilize these data to determine the presence of aldehyde and/or imine functional groups in the compounds within in 80–6000 cm⁻¹ spectral window. Additionally, the spectroscopic dataset is expected to contribute valuable information aiming at the characterization of spectra in the solid state in a broad spectral window covering fingerprint and high-wavenumber spectral region (Fig. 5).

## 3 Data Description

Table 1 shows CAS number, chemical name, molecular formula, purity and lot number of the amine and the five aldehydes used for the synthesis of the Schiff bases, whereas Table 2 contains the lot number, colour and date of synthesis of the Schiff bases.

**Table 1:** Information on starting materials.

CAS Reg. Number.	Chemical Names	Molecular Formula	Purity (%)	LOT numbers
137-07-5	2-Aminothiophenol	C_6_H_7_NS	99	ATP-A1
90-02-8	Salicylaldehyde	C_7_H_6_O_2_	98	SAL_C1
135-02-4	2-Methoxybenzaldehyde	C_7_H_7_O_2_	NA	MBZ-C2
708-06-5	2-hydroxy-1-naphthaldehyde	C_11_H_8_O_2_	99	HNT_C3
1121-60-4	2-pyridinecarboxaldehyde	C_6_H_5_NO	99	PCB_C4
98-03-3	2-thiophenecarboxaldehyde	C_5_H_4_OS	98	TCB_C5

**Table 2:** Information on synthesized Schiff bases.

Lot No.	Color	Date of Synthesis
ATP_SAL_L1	Yellow	15/01/2024
ATP_MBZ_L2	Yellow	18/01/2024
ATP-HNT_L3	Yellow	23/01/2024
ATP_PCB_L4	Light Brown	24/01/2024

All compounds feature literature MIR information by manually searching the CAS numbers using the substance search. This Reaxys assessment is based on data available in April 2025.

The IR wavenumbers and Relative IR intensity of the compounds listed in Table 1, Table 2 are shown between Table 3 and Table 12, respectively. The Relative IR Intensity is labelled as vs, s, m, w, vw and vvw to denote a very strong, strong, medium, weak, very weak and very very weak intensities. The assignment of Relative IR Intensity was achieved by using the VLOOKUP function in Microsoft Excel. VLOOKUP was also used to search and assign the IR peaks in the Excel spreadsheet (Table 3, Table 4, Table 5, Table 6, Table 7, Table 8, Table 9, Table 10, Table 11, Table 12).

**Table 3:** FTIR spectral data of ATP-A1.

Wavenumber (cm⁻¹)	Relative IR Intensity
5027	Vvw
4755	Vvw
4638	Vvw
4521	Vvw
4087	Vvw
3899	Vvw
3647	Vvw
3627	Vvw
3445	Vvw
3358	W
3188	Vvw
3063	Vvw
3017	Vvw
2874	Vvw
2779	Vvw
2617	Vvw
2524	Vvw
2328	Vvw
2115	Vvw
1901	Vvw
1780	Vvw
1605	S
1475	S
1446	W
1303	M
1249	Vvw
1157	W
1141	Vvw
1088	Vvw
1049	Vvw
1024	Vw
967	Vvw
909	Vvw
876	Vvw
849	Vvw
832	Vvw
800	Vvw
744	Vs
710	Vvw
674	Vvw
554	Vvw
532	Vvw
468	Vvw
456	Vvw
435	W
304	Vvw
260	Vvw
188	Vvw
174	Vvw
165	Vvw
152	Vvw
144	Vvw
124	Vvw
118	Vvw
101	W

**Table 4:** FTIR spectral data of SAL_C1.

Wavenumber (cm⁻¹)	Relative IR Intensity
3181	Vvw
3109	Vvw
3060	Vvw
2846	Vvw
2750	Vvw
2503	Vvw
2349	Vvw
1805	Vvw
1768	Vvw
1715	Vvw
1661	Vs
1643	Vw
1619	W
1579	W
1519	Vvw
1485	W
1458	M
1417	Vvw
1385	W
1352	Vvw
1321	Vvw
1273	S
1226	Vw
1201	Vvw
1187	W
1149	M
1113	Vw
1028	W
1028	W
1028	W
1028	W
1028	W
1028	W
1028	W
984	Vvw
946	Vvw
882	M
864	Vvw
755	W
704	Vw
664	S
607	Vvw
562	W
538	W
451	W
432	Vvw
410	W
294	Vw
262	W
216	Vw
177	Vvw
167	Vvw
150	Vvw
141	Vvw
117	Vvw
110	Vvw

**Table 5:** FTIR spectral data of MBZ-C2.

Wavenumber (cm⁻¹)	Relative IR Intensity
3337	Vvw
3071	Vvw
3037	Vvw
3016	Vvw
2976	Vvw
2946	Vvw
2927	Vvw
2865	Vw
2847	Vvw
2765	Vvw
1942	Vvw
1824	Vvw
1724	Vvw
1681	M
1663	Vw
1621	Vvw
1596	Vs
1580	Vvw
1482	M
1468	Vw
1451	Vvw
1442	Vvw
1431	W
1393	M
1302	Vvw
1283	M
1240	S
1190	M
1179	Vvw
1163	Vvw
1152	W
1102	W
1040	W
1019	M
991	Vvw
955	Vvw
873	Vvw
836	M
784	Vvw
766	S
647	M
605	Vvw
581	Vw
531	Vvw
479	M
440	Vw
401	Vvw
275	W
203	Vvw
166	Vvw
151	Vvw
121	W
100	Vw

**Table 6:** FTIR spectral data of HNT_C3.

Wavenumber (cm⁻¹)	Relative IR Intensity
3076	Vvw
3056	Vvw
2889	Vw
2803	Vvw
2347	Vvw
2114	Vvw
1812	Vvw
1702	Vvw
1620	W
1588	Vvw
1518	Vvw
1510	Vvw
1463	M
1437	Vvw
1422	Vvw
1399	W
1368	Vvw
1357	Vvw
1346	Vvw
1311	M
1273	Vvw
1245	W
1215	Vvw
1177	Vw
1162	M
1142	Vvw
1090	Vvw
1074	Vvw
1036	Vvw
1006	Vvw
988	Vvw
968	Vvw
950	Vvw
863	W
839	Vvw
793	W
775	Vvw
744	Vs
714	W
677	Vvw
653	Vw
597	Vvw
530	W
501	M
478	M
435	Vw
411	Vw
332	W
246	Vvw
226	M
202	Vvw
172	Vw
130	M
117	Vvw
104	Vw
93	Vvw
84	Vvw

**Table 7:** FTIR spectral data of PCB_C4.

Wavenumber (cm⁻¹)	Relative IR Intensity
3627	Vvw
3410	Vvw
3055	Vvw
3011	Vvw
2839	Vvw
2821	Vvw
2711	Vvw
2361	Vvw
1708	Vs
1664	Vvw
1607	Vvw
1584	M
1570	Vvw
1470	Vvw
1438	Vw
1365	Vw
1299	Vvw
1261	Vvw
1212	M
1150	Vvw
1089	Vvw
1041	Vw
994	W
905	Vvw
831	M
762	S
662	M
612	M
450	Vvw
406	M
221	M
158	Vvw
123	Vw
98	Vvw
91	Vvw

**Table 8:** FTIR spectral data of TCB_C5.

Wavenumber (cm⁻¹)	Relative IR Intensity
3947	Vvw
3834	Vvw
3535	Vvw
3307	Vvw
3089	Vvw
2836	Vvw
2820	Vvw
2790	Vvw
2753	Vvw
2711	Vvw
2652	Vvw
2336	Vvw
1876	Vvw
1669	Vvw
1655	Vs
1518	W
1417	S
1390	Vvw
1355	Vw
1336	Vvw
1326	Vvw
1234	Vw
1211	S
1148	Vvw
1081	Vvw
1045	M
997	Vvw
917	Vvw
863	W
846	Vvw
816	Vvw
756	Vvw
723	M
661	S
607	Vvw
584	Vvw
565	Vw
468	M
271	W
179	W
149	Vvw
116	Vvw
90	Vvw

**Table 9:** FTIR spectral data of ATP_SAL_L1.

Wavenumber (cm⁻¹)	Relative IR Intensity
3251	M
3055	Vvw
2115	Vvw
1785	Vvw
1614	Vvw
1583	M
1486	Vw
1475	Vvw
1455	M
1406	Vvw
1387	Vvw
1362	Vvw
1345	Vvw
1315	Vvw
1270	Vvw
1251	Vvw
1236	Vvw
1219	S
1197	Vvw
1179	Vvw
1149	Vvw
1122	Vvw
1104	Vvw
1069	Vvw
1035	Vvw
1024	Vw
972	Vw
933	Vw
922	Vvw
872	Vvw
860	Vw
817	W
752	Vvw
738	Vs
698	Vvw
671	Vvw
661	Vvw
637	Vvw
605	Vvw
574	Vvw
547	Vvw
538	Vvw
518	Vvw
498	Vvw
456	Vw
423	W
423	W
363	Vvw
326	Vvw
266	Vvw
243	Vvw
210	W
178	Vvw
139	Vvw
118	W
87	Vvw

**Table 10:** FTIR spectral data of ATP_MBZ_L2.

Wavenumber (cm⁻¹)	Relative IR Intensity
3393	Vvw
3064	Vvw
3011	Vvw
2957	Vvw
2933	Vvw
2900	Vvw
2835	Vvw
2538	Vvw
2325	Vvw
2114	Vvw
1598	Vvw
1573	W
1487	Vvw
1476	Vw
1460	M
1436	Vvw
1387	Vvw
1357	W
1307	Vvw
1279	W
1258	Vvw
1237	Vs
1190	Vvw
1172	Vvw
1158	W
1114	Vvw
1100	W
1040	Vvw
1025	M
977	Vvw
954	Vvw
909	Vvw
860	Vvw
840	Vvw
817	Vvw
803	Vvw
756	S
738	W
714	Vvw
696	Vvw
643	Vw
613	Vvw
589	Vvw
557	Vvw
530	Vw
490	Vvw
475	Vvw
460	Vvw
443	Vvw
418	Vvw
396	Vvw
352	Vvw
326	Vvw
290	Vvw
272	Vvw
231	Vvw
198	Vvw
171	Vvw
150	Vvw
130	Vvw
104	Vvw
90	Vw

**Table 11:** FTIR spectral data of ATP-HNT_L3.

Wavenumber (cm⁻¹)	Relative IR Intensity
3259	Vvw
3053	Vvw
3019	Vvw
2663	Vvw
2346	Vvw
1759	Vvw
1621	Vw
1599	Vw
1583	Vvw
1555	M
1521	Vvw
1460	M
1442	Vvw
1398	Vvw
1363	Vvw
1321	Vw
1299	Vvw
1267	Vw
1242	Vvw
1219	W
1190	Vvw
1178	Vvw
1161	W
1139	Vvw
1139	Vvw
1120	Vvw
1082	Vw
1054	Vvw
1039	Vvw
1017	Vvw
966	Vvw
943	Vw
917	Vvw
879	Vvw
879	Vvw
859	Vw
851	Vvw
817	M
793	Vvw
777	Vvw
768	Vvw
756	Vw
742	Vs
710	Vvw
692	Vvw
642	Vw
613	Vvw
581	Vw
561	Vvw
547	Vvw
533	Vvw
516	W
482	Vvw
467	Vvw
449	Vw
437	Vvw
418	W
365	W
365	W
319	Vvw
308	W
308	W
245	Vw
245	Vw
225	M
204	Vvw
173	Vvw
151	W
134	Vvw
116	Vw
95	Vvw

**Table 12:** FTIR spectral data of ATP_PCB_L4.

Wavenumber (cm⁻¹)	Relative IR Intensity
3052	Vw
3005	Vvw
2325	Vvw
2115	Vvw
1663	Vvw
1585	W
1565	Vw
1510	Vw
1456	W
1433	Vs
1318	W
1295	Vvw
1266	Vvw
1235	Vw
1154	Vvw
1090	Vvw
1070	Vvw
1049	Vvw
1014	Vvw
996	W
979	M
965	Vvw
936	Vvw
899	Vw
858	Vw
781	M
771	Vvw
758	M
739	S
728	W
703	Vw
681	Vvw
619	M
572	Vvw
551	Vw
466	W
425	W
400	M
344	Vvw
313	W
278	Vvw
211	W
179	Vvw
151	Vvw
138	Vvw
108	Vvw
88	Vvw

The primary amine spectrum is shown in Fig. 1, while the aldehydes spectra are shown between Fig. 2, Fig. 3, Fig. 4, Fig. 5, Fig. 6. Meanwhile, Schiff bases spectra are shown between Fig. 7, Fig. 8, Fig. 9, Fig. 10.

**Fig. 1:**
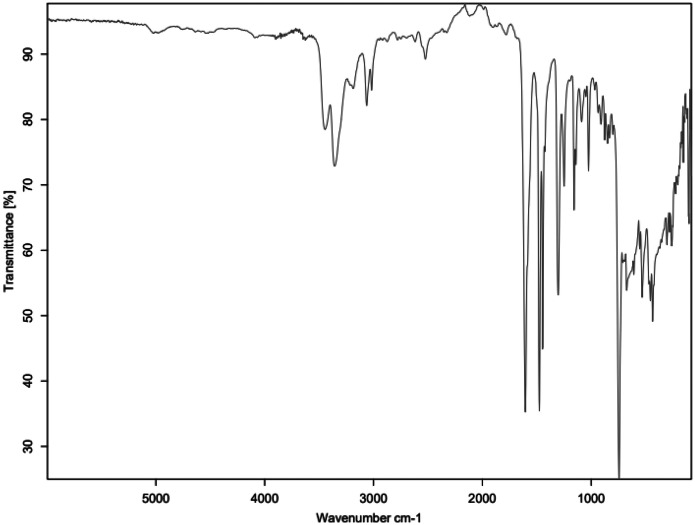
FTIR for ATP-A1.

**Fig. 2:**
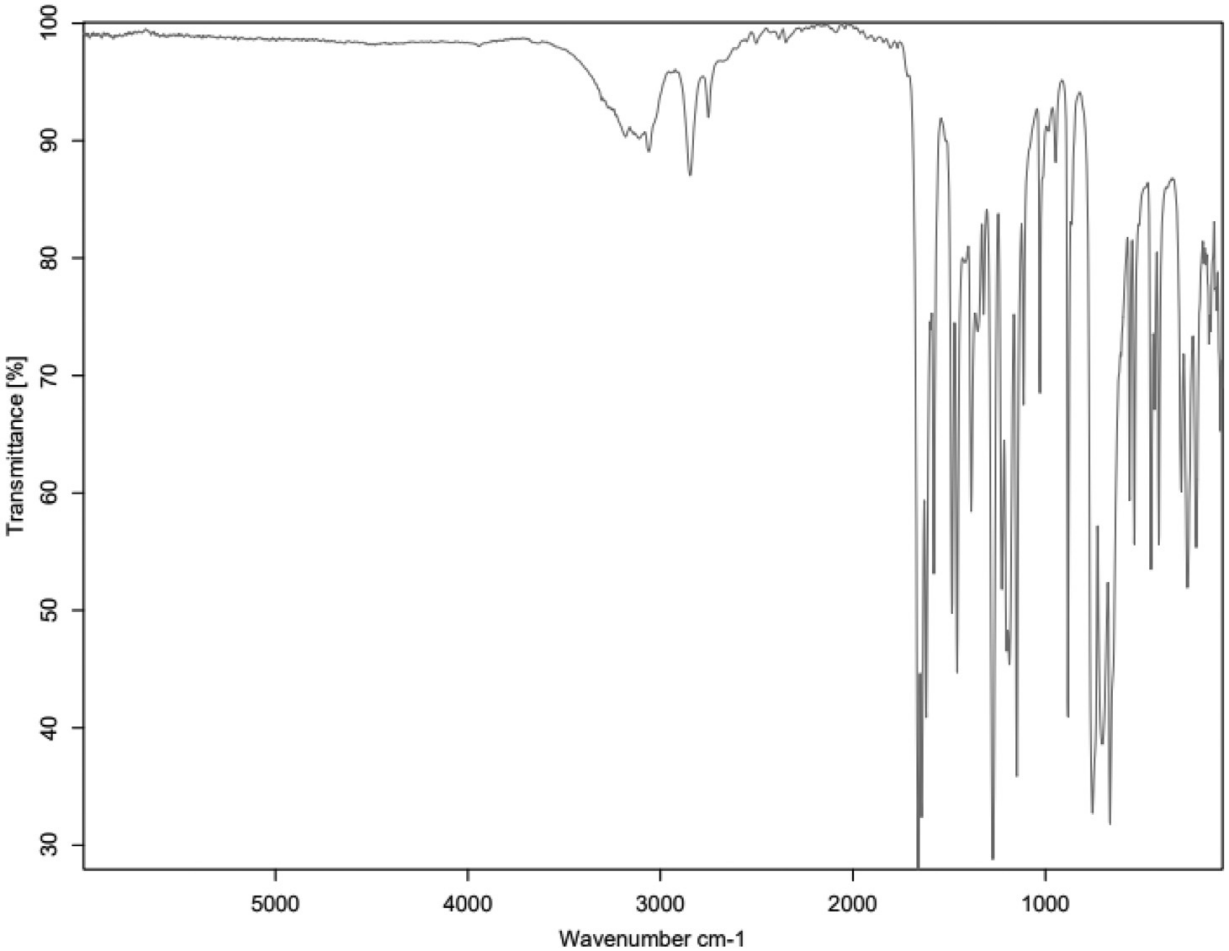
FTIR for SAL_C1.

**Fig. 3:**
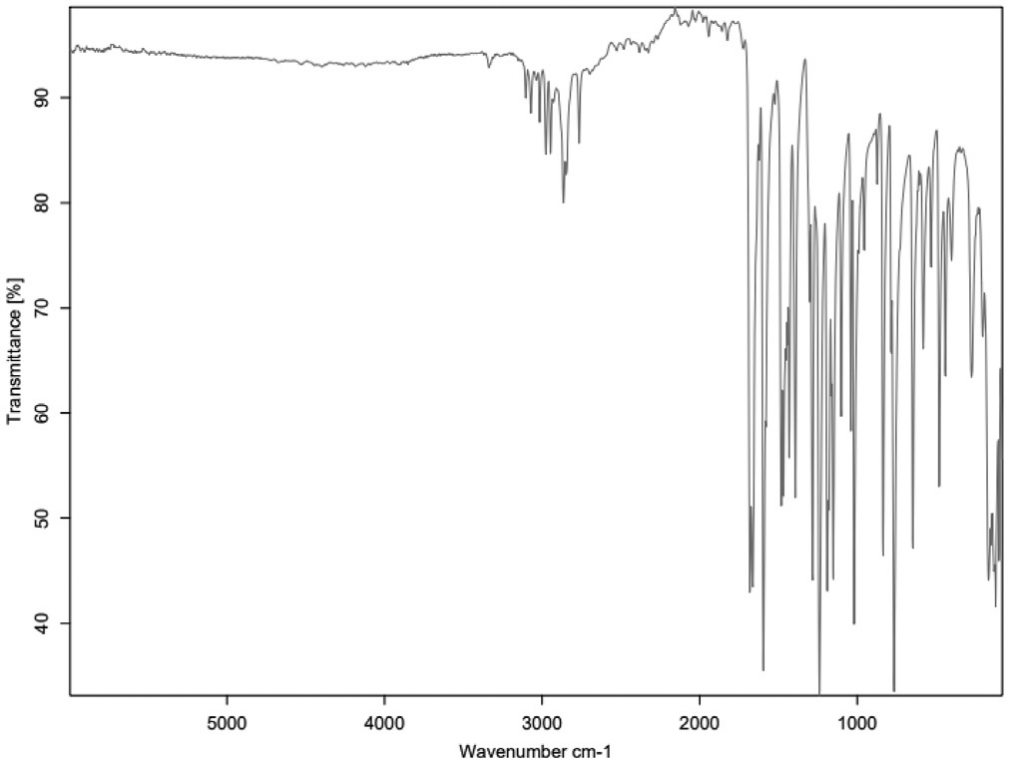
FTIR for MBZ-C2.

**Fig. 4:**
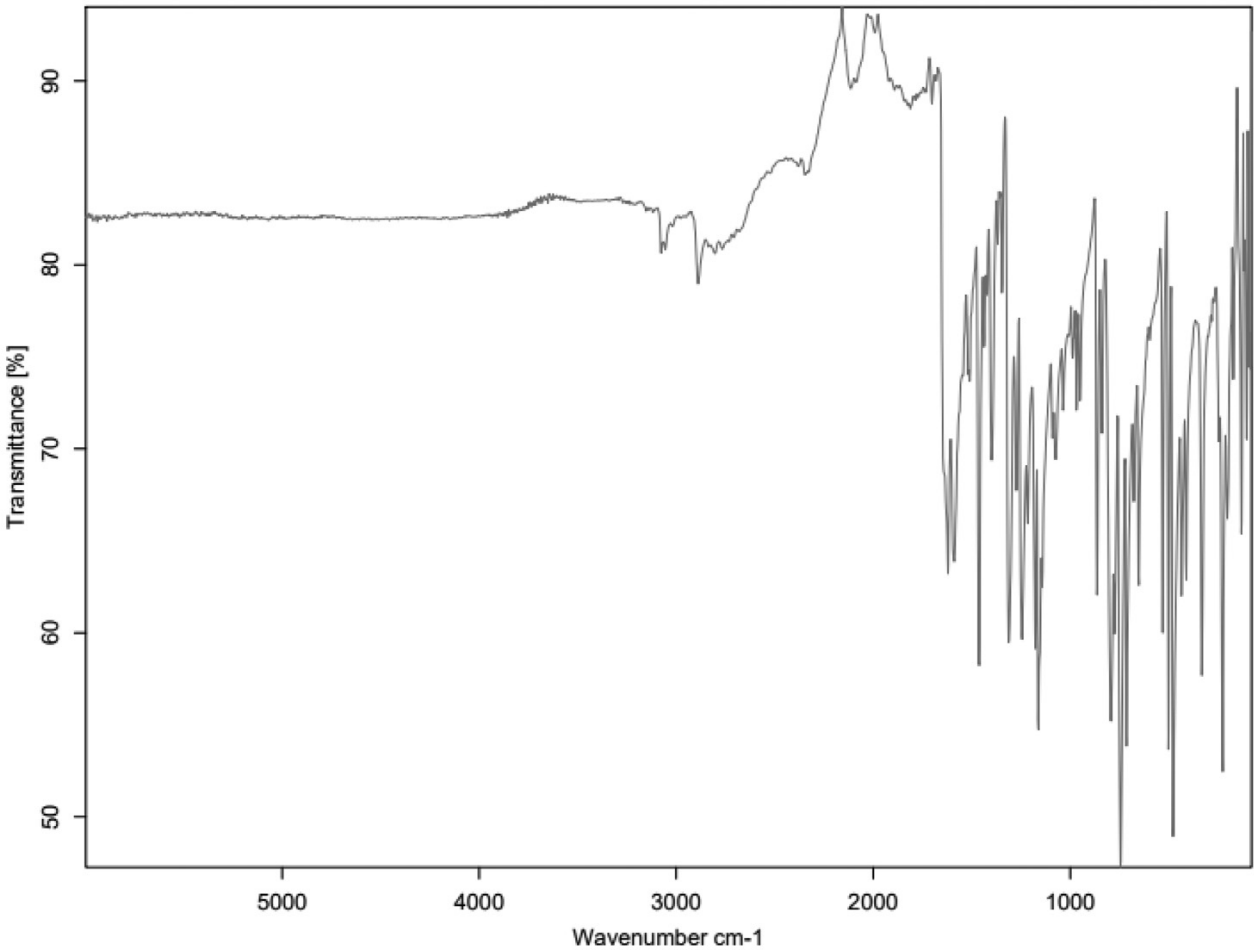
FTIR for HNT_C3.

**Fig. 5:**
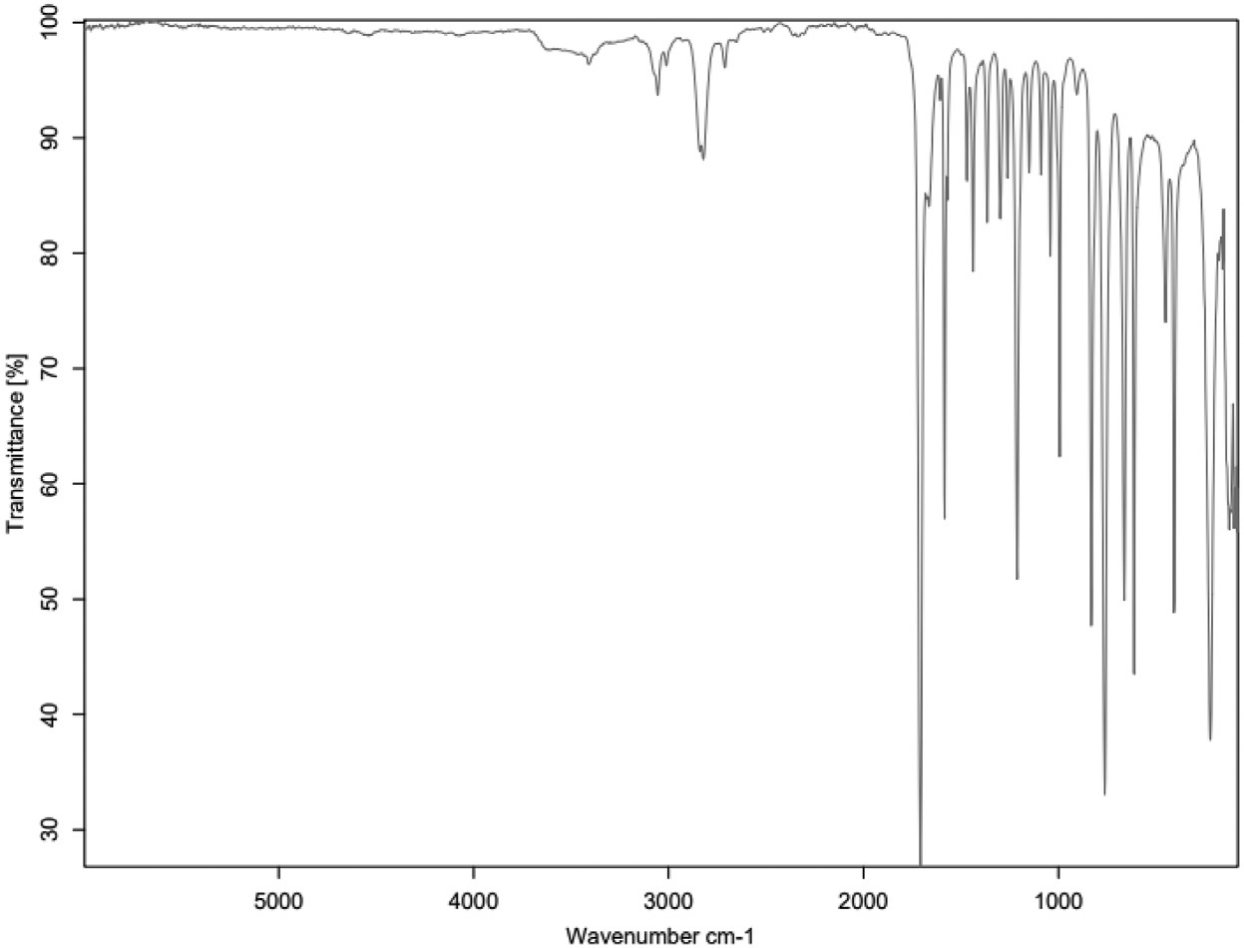
FTIR for PCB_C4.

**Fig. 6:**
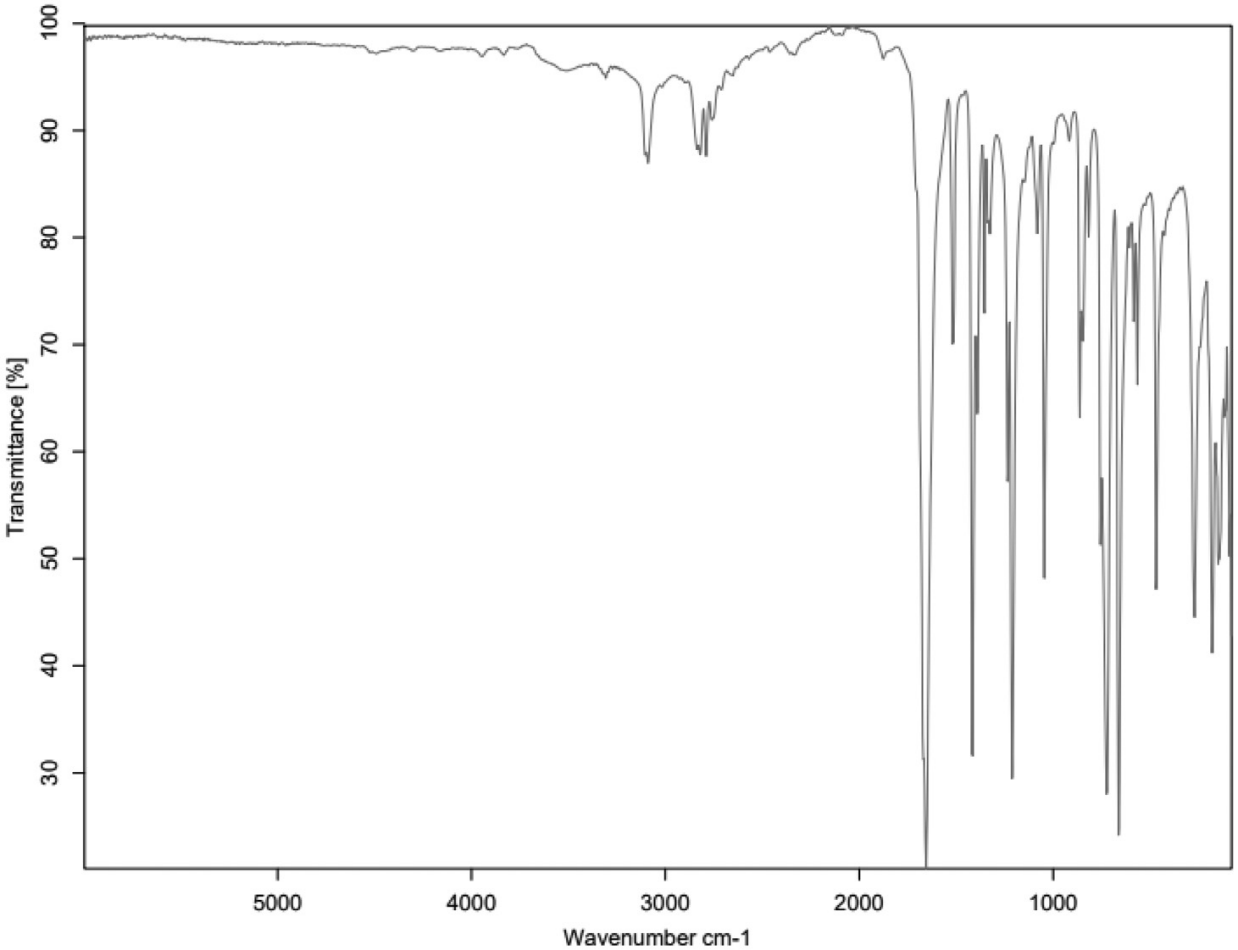
FTIR for TCB_C5.

**Fig. 7:**
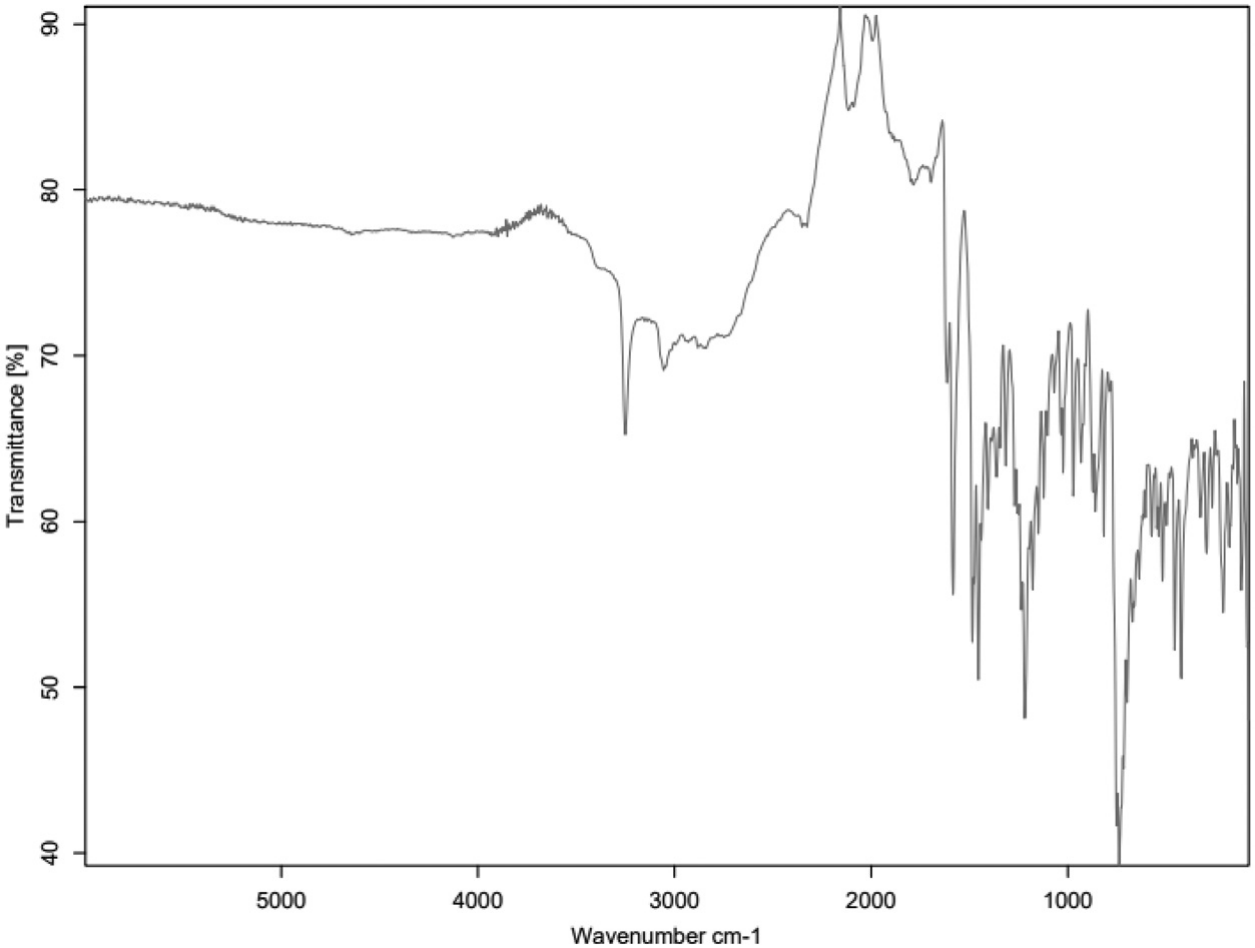
FTIR for ATP_SAL_L1.

**Fig. 8:**
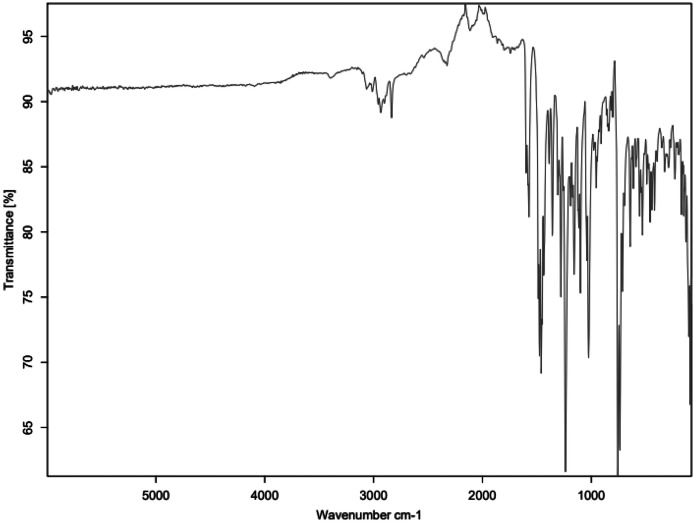
FTIR for ATP_MBZ_L2.

**Fig. 9:**
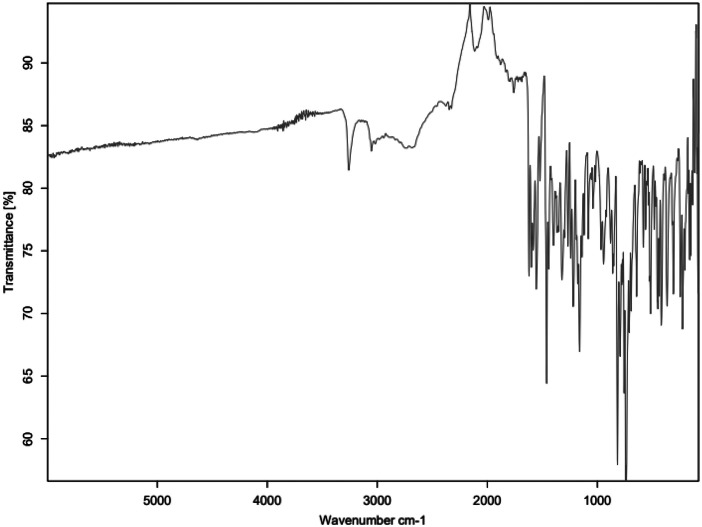
FTIR for ATP-HNT_L3.

**Fig. 10:**
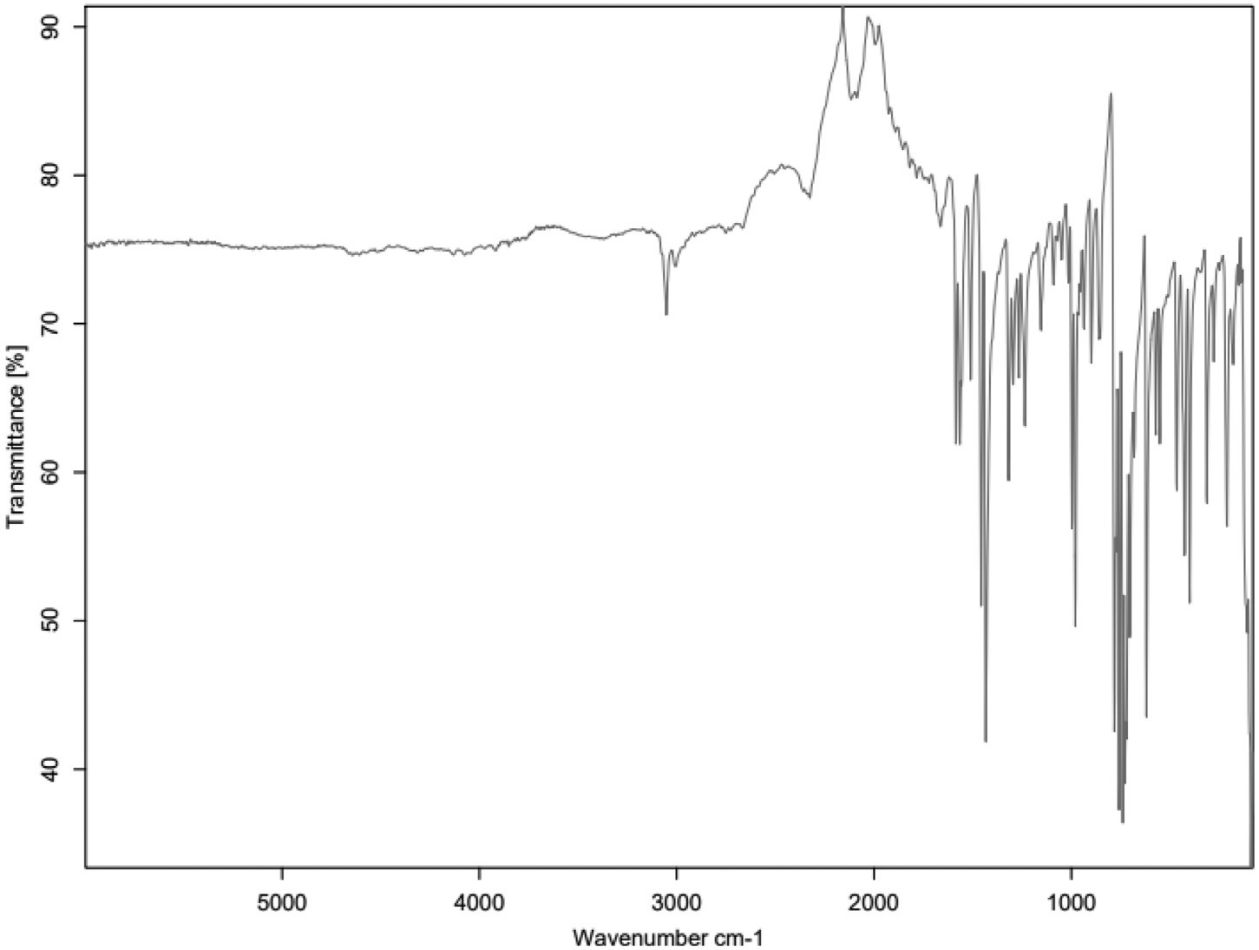
FTIR for ATP_PCB_L4.

## 4 Experimental design

**Fig. 11:**
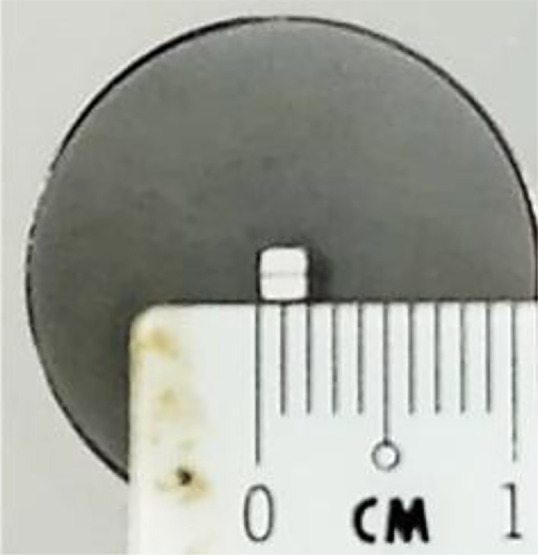
ATR diamond size of INVENIO-R (2 mm).

**Fig. 12:**
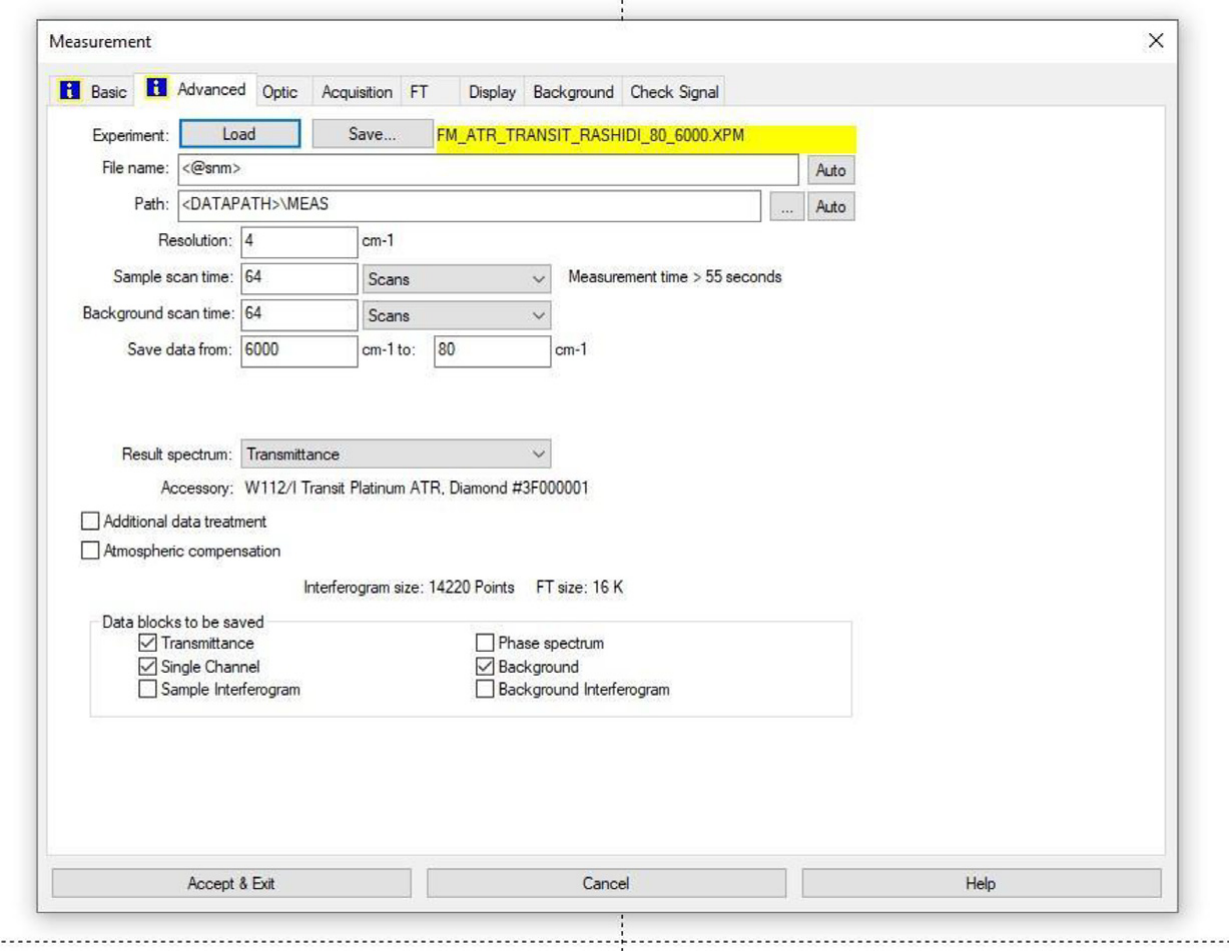
Configurations and numerical values of the advanced parameters (such as resolution, sample scan time, background scan time, and spectral range).

**Fig. 13:**
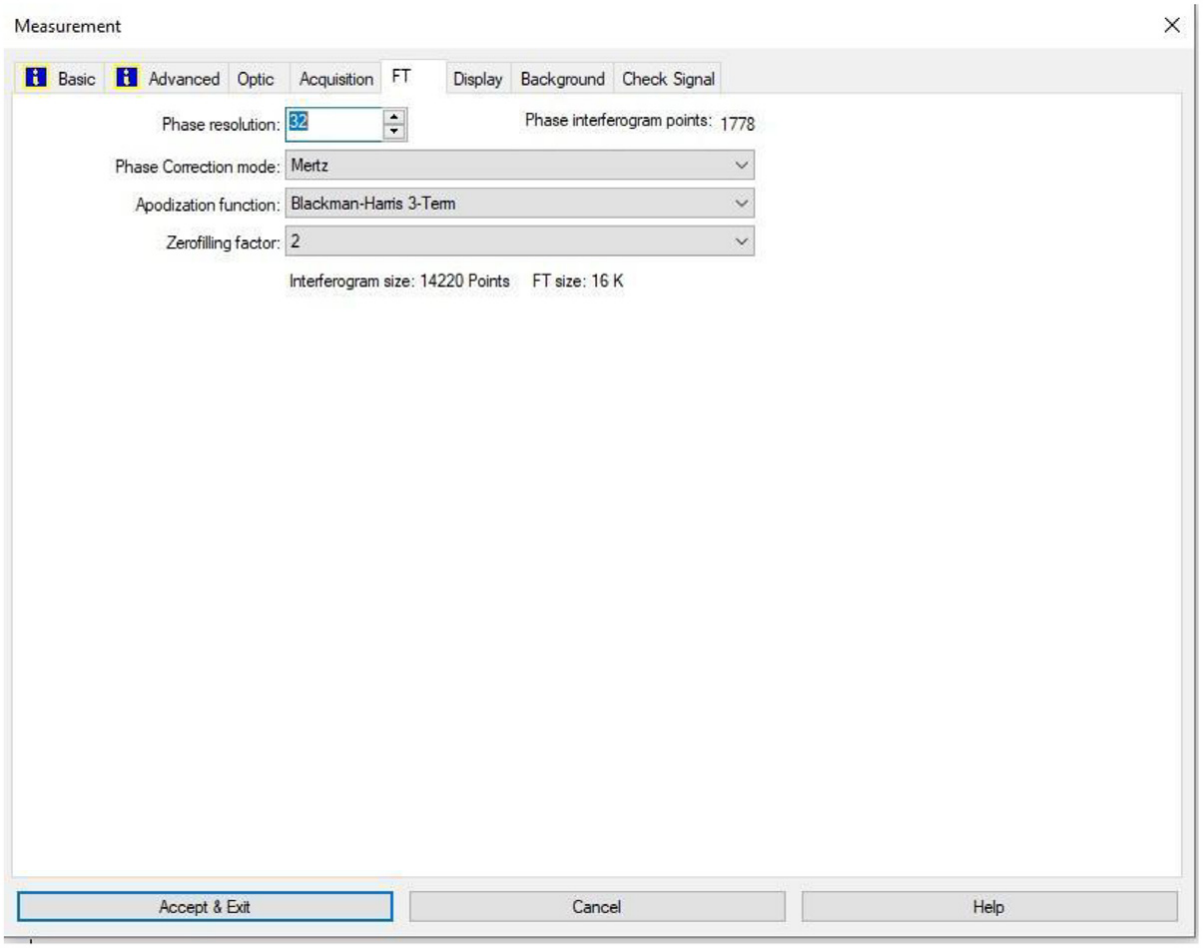
Configurations and numerical values of the phase resolution at 32 stored within the Fourier transform.

**Fig. 14:**
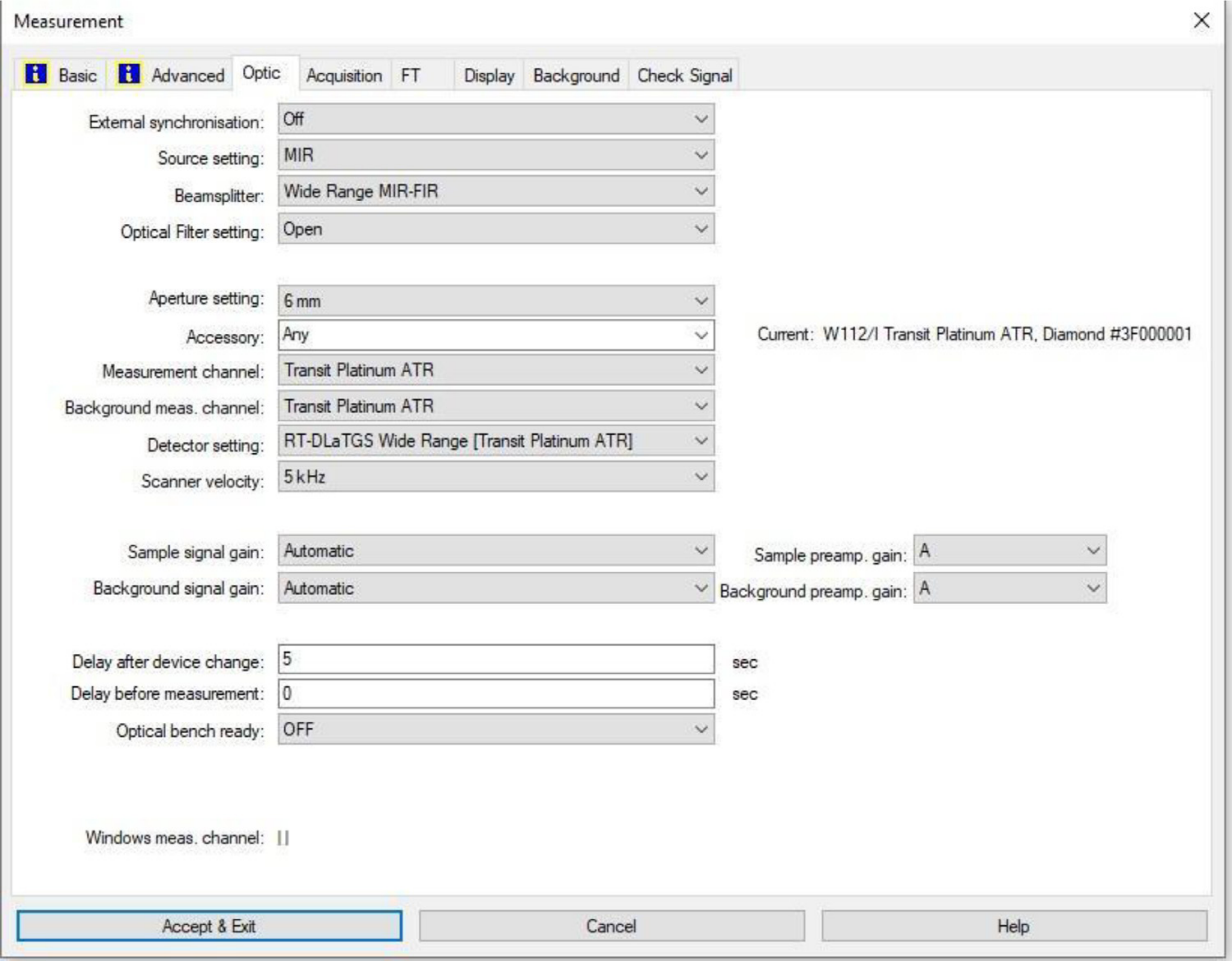
Configurations and numerical values of the optical parameters used.

The FTIR transmittance (%) spectra of each sample analysed were acquired between 80 and 6000 cm⁻¹ covering the NIR, MIR, and FIR spectral regions, using a Bruker Invenio-R (Universiti Putra Malaysia, Malaysia) spectrometer equipped with an ATR (2 mm) diamond, as shown in Fig. 11. Spectra acquisition involved 64 scans with a spectral resolution of 4 cm⁻¹. The FTIR spectra were processed using OPUS 8.7.41 software (Fig. 12) and then stored in the Mendeley depository. A temperature control unit was used to maintain a constant temperature of ∼26 °C during the acquisition of the IR spectra. Before measuring the spectrum of each sample, the ATR was wiped with acetone to remove contaminants from the previous sample and allowed to evaporate to dryness. A background spectrum was collected to subtract any unwanted residual peaks from the sample spectrum and to avoid contaminants reading. Then, the sample spectra were recorded immediately on the ATR and analysed by OPUS 8.7.41. The configurations and numerical values of the advanced parameters (such as resolution, sample scan time, background scan time, and spectral range) are saved [10]. Additionally, the phase resolution at 32 is stored within the Fourier transform (Fig. 13) and the optical parameters are also displayed under these experimental conditions (Fig. 14).

## Limitations

Not applicable.

## Ethics Statement

The work does not involve human subjects, animal experiments, or data collected from social media platforms.

## Credit Author Statement

**Mohd Rashidi Abdull Manap:** Writing-original draft, Supervision, Funding acquisition, Software; **Umar Sani:** Conceptualization, Funding acquisition, Methodology, Writing-original draft, Supervision; **Musbahu Yahaya:** Conceptualization, Methodology, Validation, Writing-original draft, Project administration; **Ahmad Zaidi Ismail:** Methodology, Software, Investigation, Visualization; **Qhurratul Aina Kholili:** Writing-original draft, Software; **Fatin Abu Hasan:** Writing-original draft, Software; **Siti Nurul Ain Md Jamil:** Writing-original draft; **Reda Dzingelevičienė:** Writing-original draft; **Saiful Saiful:** Writing-original draft, **Giorgio S. Senesi:** Writing-original draft, **Herculano Martinho:** Writing-original draft.

## Data Availability

Mendeley DataExperiment files and measurement parameters for Bruker Invenio-R (Original data)
Mendeley DataInfrared spectra of primary amine and some aldehydes with their corresponding Schiff base obtained using Invenio-R spectrometer. 1 (Original data)
